# Polyketides From the Endophytic Fungus *Cladosporium* sp. Isolated From the Mangrove Plant *Excoecaria agallocha*

**DOI:** 10.3389/fchem.2018.00344

**Published:** 2018-08-14

**Authors:** Liping Wang, Xiuli Han, Guoliang Zhu, Yi Wang, Arthit Chairoungdua, Pawinee Piyachaturawat, Weiming Zhu

**Affiliations:** ^1^State Key Laboratory of Functions and Applications of Medicinal Plants, Guizhou Medical University, Guiyang, China; ^2^Key Laboratory of Marine Drugs, Ministry of Education of China, School of Medicine and Pharmacy, Ocean University of China, Qingdao, China; ^3^College of Life Sciences, Shandong University of Technology, Zibo, China; ^4^Department of Physiology, Faculty of Science, Mahidol University, Bangkok, Thailand; ^5^Laboratory for Marine Drugs and Bioproducts of Qingdao National Laboratory for Marine Science and Technology, Qingdao, China

**Keywords:** *Cladosporium* sp., mangrove fungus, *Excoecaria agallocha*, polyketides, anti-oxidation

## Abstract

Five new polyketides (**2**–**6**) and ten known compounds (**1** and **7**–**15**) were obtained from the fermentation products of the endophytic fungus *Cladosporium* sp. OUCMDZ-302 with the mangrove plant, *Excoecaria agallocha* (Euphorbiaceae). The new structures of **2**–**6** were established on the basis of ECD, specific rotation and spectroscopic data as well as the chemical calculation. Compound **7** showed cytotoxicity against H1975 cell line with an IC_50_ value of 10.0 μM. Compounds **4** and **8**–**10** showed radical scavenging activity against DPPH with the IC_50_ values of 2.65, 0.24, 5.66, and 6.67 μM, respectively. In addition, the absolute configuration of compound **1** was solidly determined by X-ray and sugar analysis of the acidic hydrolysates for the first time as well as those of compounds **8**–**10** in this paper.

## Introduction

Mangrove plants and endophytic fungi are two principal sources of new and bioactive natural products (Zhang et al., 2006; Wu et al., 2008). *Excoecaria agallocha* (Euphorbiaceae), also known as blind-your-eye, is mainly used to treat sores and stings. More than 72 cytotoxic diterpenoids have been identified from *E*. *agallocha*, structurally belonging to labdane (Konishi et al., 1999, 2003; Anjaneyulu and Rao, 2000; Annam et al., 2015), isopimarane/*ent*-isopimarane (Anjaneyulu et al., 2003; Wang and Guo, 2004; Kang et al., 2005; Wang et al., 2005; Gowri Ponnapalli et al., 2013), atisane/*ent*-atisane (Konishi et al., 2000; Wang et al., 2009), *ent*-kaurane (Anjaneyulu et al., 2002; Li et al., 2010), and beyerane-type (Anjaneyulu et al., 2002).

**Figure 1:**
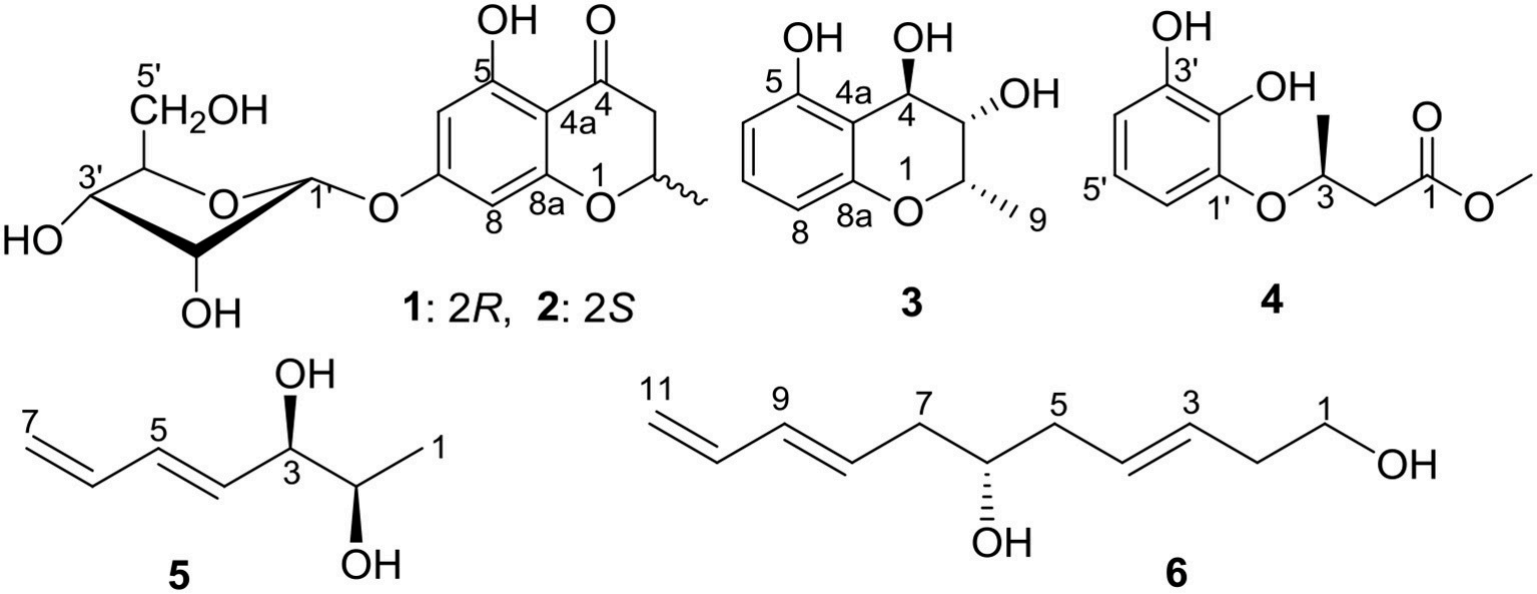
The structure of compounds **1–6**.

In our ongoing investigations of new and bioactive compounds from endophytes associated with mangrove plants (Lin et al., 2008; Lu et al., 2009, 2010; Wang et al., 2012; Kong et al., 2014; Zhu et al., 2018), an endogenous fungal strain OUCMDZ-302 identified as *Cladosporium* sp., was isolated from the surface-sterilized stems of *E*. *agallocha*. The secondary metabolites of the genus *Cladosporium* were mainly reported as polyketides derivatives, such as macrolides (Jadulco et al., 2001; Zhang et al., 2001; Shigemori et al., 2004), α-pyrones (Jadulco et al., 2002), α-pyridone (Ye et al., 2005), and binaphthyl derivatives (Sakagami et al., 1995). Herein we report five new polyketides (**2**–**6**) (Figure 1) isolated from the EtOAc extract of *Cladosporium* sp. OUCMDZ-302, along with the ten known structures (Figure S36 and Table S1), (2*R*)-7-*O*-α-D-ribofuranosyl-5-hydroxy**-**2-methylchroman-4-one (**1**) (Hu et al., 2017), 7-*O*-α-D-ribosyl-5-hydroxy-2-propylchromone (**7**) (Zhao et al., 2015), 3-(2,3-dihydroxy phenoxy)butanoic acid (**8**) (Dai et al., 2009), (2*S*,4*S*)-4-methoxy-2-methylchroman-5-ol (**9**) (Wu et al., 2010), (2*S*,4*S*)-2-methylchroman-4,5-diol (**10**) (Teles et al., 2005), (±)-5,7-dihydroxy-2-methyl chroman-4-one (**11**) (Rao et al., 1994), (±)-5-hydroxy-2-methylchroman-4-one (**12**) (Dai et al., 2006), 1-(2,6-dihydroxyphenyl) ethanone (**13**) (Dhami and Stothers, 1965), 1-(2,6-dihydroxyphenyl)-1-butanone (**14**) (Huang et al., 2005), and 2-butyryl-3,5-dihydroxycyclohex-2-enone (**15**) (Igarashi et al., 1993). Compound **7** showed inhibitory activity against H1975 cell line with an IC_50_ value of 10.0 μM. Compounds **4** and **8**–**10** exhibited radical scavenging activity against DPPH with IC_50_ values of 2.65, 0.24, 5.66, and 6.67 μM, respectively. In addition, the absolute configurations of compounds **8**–**10** were resolved and that **1** was solidified in this paper.

## Materials and methods

### General experimental procedures

The NMR, ECD, [α]_D_, UV and IR spectra were recorded on JEOL JNM-ECP 600, JASCO J-810, JASCO P-1020 digital, Beckman DU® 640 and Nicolet NEXUS 470 spectrophotometers, respectively. ESI-MS, EI-MS and GC-MS were measured on Q-TOF ULTIMA GLOBAL GAA076 LC, VG Autospec-3000 and Agilent 6890/5973 spectrometers, respectively. Semipreparative HPLC and chiral separation was performed on a YMC-pack ODS-A column [10 × 250 mm, 5 μm, 4 mL/min] and a CHIRALPAK IA column [20 × 250 mm, 5 μm, 10 mL/min]. TLC was performed on plates precoated with silica gel GF254 (10–40 μm). The column chromatography (CC) was performed over silica gel (200–300 mesh, Qingdao Marine Chemical Factory, Qingdao, China) and Sephadex LH-20 (Amersham Biosciences, Sweden), respectively. The seawater for the cultural medium of *Cladosporium* sp. OUCMDZ-302 was collected from Yellow Sea near Qingdao.

### Fungal material

The strain *Cladosporium* sp. OUCMDZ-302 was isolated from the surface sterilized stems of the mangrove plant *E. agallocha* grown in Wenchang, Hainan, China. Briefly, the stems were washed with tap water and sterile distilled water in sequence. The stems with clean surface were further sterilized in a sequence of 75% ethanol for 2 min, 0.1% of HgCl_2_ for 3 min, and sterile distilled water. The outer bark was removed, and the inner bark was cut into small pieces that were then placed on a potato dextrose agar (PDA) plate and cultured at 28°C for 3 days. A single colony was transferred to PDA media and was identified according to its morphological characteristics (Figure S35) by Prof. Kui Hong, Wuhan University. A voucher specimen is deposited in our laboratory at −80°C. The working strain was prepared on PDA slants and stored at 4°C.

### Fermentation and extraction

The producing fungal strain *Cladosporium* sp. OUCMDZ-302 was inoculated into a 500 mL cylindrical flask containing 100 mL of seawater consisting of 2% maltose, 2% mannitol, 1% glucose, 1% monosodium glutamate, 0.3% yeast extract, 0.1% corn flour, 0.05% KH_2_PO_4_, 0.03% MgSO_4_· 7H_2_O (pH 6.5) and cultured at 28°C for 48 h on a rotary shaker at 120 rpm. The seed culture was transferred into three hundred and fifty 500 mL conical flasks (200 mL/flask) containing the same medium, and performed at 28°C for 7 days on rotary shakers at 160 rpm. The whole fermentation broth (70 L) was filtered through cheese cloth to separate the mycelia from filtrate. The filtrate was concentrated to about one-quarter of the original volume under reduced pressure and then extracted three times with equal volumes of ethyl acetate (EtOAc) and concentrated to dryness. The mycelia were extracted three times with acetone and concentrated to an aqueous solution. The aqueous solution was subsequently extracted three times with equal volumes of EtOAc and concentrated. Both EtOAc extractions were combined to give 45 g of the extract.

### Isolation

The extract (45 g) was separated into eight fractions (Fr.1–Fr.8) on a silica gel column (8.5 × 15 cm, 200–300 mesh) using a step gradient elution with CHCl_3_-petroleum ether (V/V 0:100–100:0, 4 L) and then MeOH–CHCl_3_ (V/V 0:100–100:0, 16 L). Fr.1 (5.4 g) was separated on a silica gel column (4.5 × 10 cm, 200–300 mesh) eluted with CHCl_3_-petroleum ether (V/V, 1: 1, 3L) to give **12** (1g). Fr.3 (0.3 g) was further purified by semipreparative HPLC (60% MeOH/H_2_O) to give **10** (7 mg, *t*_R_ 4.97 min). Fr.4 (4.3 g) was separated into two subfractions by column chromatography over silica gel (RP-18) eluting with gradient H_2_O-MeOH (50–100%). Fr.4-1 (1.4 g) was separated by Sephadex LH-20 (3 × 75 cm, MeOH, 300 mL) to obtain three fractions (130 mL, Fr.4-1-1; 90 mL, Fr.4-1-2; 80 mL, Fr.4-1-3). Fr.4-1-2 (140 mg) was purified by semipreparative HPLC (30% MeOH/H_2_O) to yield **5** (1 mg, *t*_R_ 8.24 min), **13** (30 mg, *t*_R_ 20.20 min), and **15** (5 mg, *t*_R_ 18.15 min). Fr.4-1-3 (190 mg) was purified by semipreparative HPLC (30% MeOH/H_2_O, 0.15% CF_3_CO_2_H) to give **4** (15 mg, *t*_R_ 16.76 min) and **8** (30 mg, *t*_R_ 14.41 min). Fr.4-2 (360 mg) was purified by semipreparative HPLC (50% MeOH/H_2_O) to give **6** (10 mg, *t*_R_ 12.28 min), and **14** (24 mg, *t*_R_ 18.12 min). Fr.5 (1.1 g) was separated into two subfractions by a silica gel column (2.6 × 10 cm, 200–300 mesh) eluted with MeOH–CHCl_3_ (V/V 1:40, 1L). Fr.5-1 (40 mg) was purified by semipreparative HPLC (25% MeOH/H_2_O) to give **3** (3 mg, *t*_R_ 6.76 min), and Fr.5-2 (80 mg) was purified by semipreparative HPLC (50% MeOH/H_2_O, 0.15% CF_3_CO_2_H) to give compounds **11** (5 mg, *t*_R_ 6.78 min). Fr.6 (2.8 g) was separated into two subfractions by a silica gel column (4.5 × 10 cm, 200–300 mesh) eluted with CHCl_3_-petroleum ether MeOH–CHCl_3_ (V/V 1:25, 2L). Fr.6-1 (110 mg) was purified by semipreparative HPLC (60% MeOH/H_2_O) to give **9** (10 mg, *t*_R_ 10.28 min). Fr.6-2 (340 mg) was purified by semipreparative HPLC (50% MeOH/H_2_O) to give **7** (18 mg, *t*_R_ 13.55 min) and the mixture of **1** and **2** (70 mg, *t*_R_ 5.56 min). The mixture of **1** and **2** were further purified by a chiral column (Chiralpak IA, MeOH–MeCN–EtOH 40:40:20) to yield compound **1** (35.4 mg, *t*_R_ 8.22 min) and **2** (23.7 mg, *t*_R_ 4.69 min).

### ECD, [α]_D_ and coupling constant calculation

Calculations for ECD and [α]_D_ were performed in HyperChem 7.5 and Gaussian 03 (Frisch et al., 2004; Chen et al., 2016; Jin et al., 2018). Karplus formula was used to compute the coupling constant (^3^*J*) from the proton-proton torsion angle (Haasnoot et al., 1980).

### Cytotoxic assays

Cytotoxicities of compounds **1**–**14** against HL-60 and K562 cell lines were assayed by the MTT method (Mosmann, 1983), while those for BEL-7402, A549, HeLa, and H1975 cell lines were tested by SRB (Skehan et al., 1990) methods. Adriamycin was used as the positive control with the IC_50_ values of 0.02, 0.21, 0.48, 1.32, 0.32, and 0.38, respectively.

### Anti-oxidant activities

The anti-oxidant activities of compounds **1**–**14** were evaluated by DPPH assay *in vitro* (Wang et al., 2007). Vitamin C was used as the positive control with an IC_50_ value of 3.29 μM.

### Antimicrobial assays

The antimicrobial activities of compounds **1**–**14** against *E*. *coli, E*. *aerogenes, P*. *aeruginosa, B*. *subtilis*, and *C*. *albicans* were evaluated by an agar dilution method (Zaika, 1988). Ciprofloxacin lactate and ketoconazole was used as the positive controls for bacteria and fungi with MIC values of 4.0, 0.5, 32.0, 16.0, 4.1 μg/mL, respectively.

## Results and discussion

### Identification of compounds

**Figure 2:**
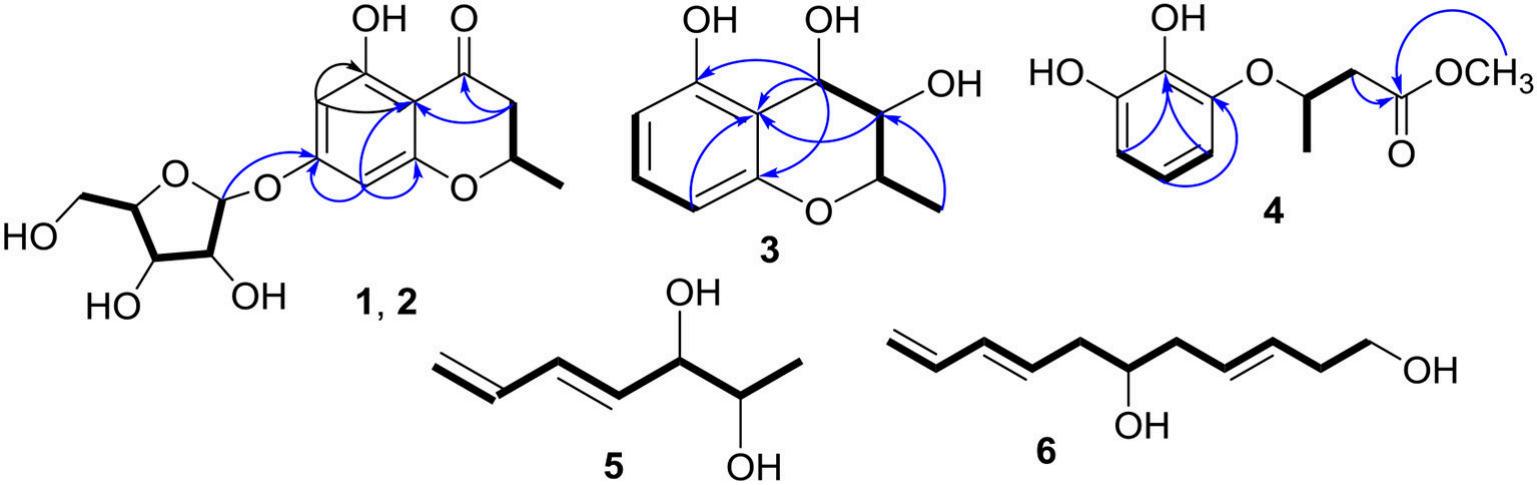
Key HMBC (→ ) and ^1^H–^1^H COSY (–) correlations of **1**–**6**.

**Figure 3:**
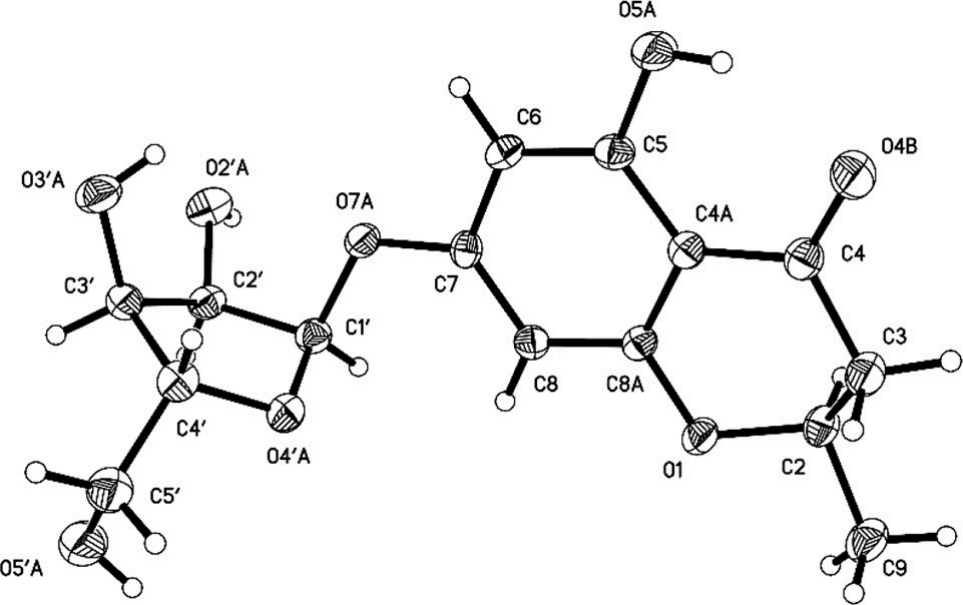
Final X-ray Drawing of compound **1**.

**Figure 4:**
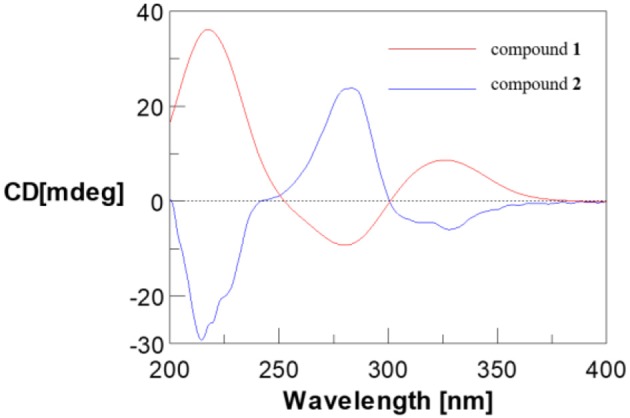
ECD curve of compounds **1** and **2**.

Compounds **1** and **2** were first isolated as an isomeric mixture whose molecular formula was determined to be C_15_H_18_O_8_ by HRESIMS at *m/z* 327.1068 [M+H]^+^ (calcd 327.1080), indicating seven degrees of unsaturation. An interpretation of the 1D (Table 1, Figures S1–S3) and 2D NMR (Figure 2 and Figures S4–S6) spectra established a pentose moiety and a benzopyrane moiety similar to those of 5,7-dihydroxy-2-methylchroman-4-one (**11**) (Rao et al., 1994). The upfield shift of C-7 (−1.5 ppm) and the key HMBC correlations between the anomeric proton ($\delta_{\text{H}-1^{\text{′}}}$ 5.66/5.68) and C-7 (δ 165.1/165.0) indicated that **1** and **2** were 7-*O*-pentosides of **11**. Acidic hydrolysis of the mixture of **1** and **2** with 2 *M* HCl yielded (±)-**11** and D-ribose that was identified by GC-MS analysis of the reaction products with L-cysteine methyl ester and Me_3_SiCl (Figures S33, S34) (Deyrup et al., 2007). These data indicated that **1** and **2** are a pair of epimers at C-2. Separation of 2-epimeric mixture of **1** and **2** was achieved on a chiral column using MeOH-MeCN-EtOH as eluent. And then, NMR data of optically-pure **1** (Figures S7, S8) and **2** (Figures S9, S10) were obtained. X-ray single crystal diffraction of **1** revealed the α-glycosidic bond and 2*R*-configuration (Figure 3). The ECD Cotton effects of compounds **1** and **2** were opposite in sign (Figure 4), confirming the opposite configuration of C-2. Thus, the structures of **1** and **2** were unambiguously elucidated as (2*R*)- and (2*S*)-7-*O*-α-D- ribofuranosyl-5-hydroxy**-**2-methylchroman-4-one, respectively. This is the first time that to solidify the absolute configuration of compound **1**, although it was reported last year (Hu et al., 2017).

**Table 1:** ^1^H (600 MHz) and ^13^C (150 MHz) NMR Data of Compounds **1**–**4** in DMSO-*d*_6_.

**No**.	**1**		**2**		**3**		**4^a^**	
	**δ_C_**	**δ_H_(*J* in Hz)**	**δ_C_**	**δ_H_(*J* in Hz)**	**δ_C_**	**δ_H_(*J* in Hz)**	**δ_C_**	**δ_H_(*J* in Hz)**
2	73.9, CH	4.61(ddq, 12.3, 6.3, 3.1)	74.0, CH	4.61(ddq, 12.3, 6.3, 3.1)	69.0, CH	4.14 dq(6.6, 0.8)	40.7, CH_2_	2.79 (dd, 16.5, 8.8) 2.63 (dd, 16.5, 3.3)
3	42.5, CH_2_	2.80 (dd,17.2, 12.3) 2.65 (dd, 17.2, 3.1)	42.6, CH_2_	2.81 (dd, 17.2,12.3) 2.64 (dd,17.2, 3.1)	69.8, CH	3.45 brs	73.4, CH	4.47 (m)
4	197.3, C		197.3, C		62.6, CH	4.48 (d, 2.2)	19.5, CH_3_	1.34 (d, 6.6)
4a	102.9, C		102.9, C		111.2, C			
5	162.6, C		162.7, C		158.3, C			
6	96.6, CH	6.11 (d, 2.2)	96.7, CH	6.11 (d, 2.2)	107.1, CH	6.18(dd, 8.3, 1.1)		
7	165.1, C		165.1, C		128.8, CH	6.91(dd,8.3, 8.2)		
8	95.6, CH	6.10 (d, 2.2)	95.6, CH	6.09 (d, 2.2)	106.8, CH	6.32(dd, 8.2, 1.1)		
8a	162.9, C		162.9, C		156.1, C			
9	20.4, CH_3_	1.40 (d, 6.3)	20.4, CH_3_	1.40 (d, 6.3)	17.5, CH_3_	1.31 (d, 6.6)		
1'	99.9, CH	5.67 (d, 4.5)	99.9, CH	5.65 (d, 4.5)			143.9, C	
2'	71.5, CH	4.07 (m)	71.5, CH	4.07(m)			136.6, C	
3'	69.2, CH	3.91 (dd, 6.3, 3.9)	69.2, CH	3.91 (m)			145.5, C	
4'	86.6, CH	3.94 (dd, 7.6, 3.9)	86.6, CH	3.94 (m)			110.7, CH	6.70 (dd, 7.7, 2.2)
5'	61.4, CH_2_	3.47 (m)	61.5, CH_2_	3.46(m)			119.0, CH	6.68 (t, 7.7)
6'							113.0, CH	6.52 (dd, 7.7, 2.2)
CH_3_O-							52.5, CH_3_	3.78 (s)

a *Measured in CDCl_3_ and δ_C−1_ was 174.0*.

**Figure 5:**
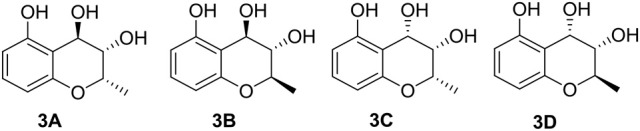
Four possible relative configurations of compound **3**.

The molecular formula of compound **3** was determined to be C_10_H_12_O_4_ based on the HRESIMS peak at *m*/*z* 195.0659 [M–H]^−^ (calcd 195.0657), indicating five degrees of unsaturation. The NMR data (Table 1, Figures S11–S14) was similar to those of **10** (Teles et al., 2005), except for the upfield methylene signal at δ_H/C_ 1.50 & 1.88/38.0 that was replaced by the one of an oxygenated methine at δ_H/C_ 3.45/69.8. This was further supported by the ^1^H-^1^H COSY from H-9 (δ 1.31) to H-4 (δ 4.48) through H-2 (δ 4.14) and H-3 (δ 3.45) (Figure 2 and Figure S15), and the key HMBC correlations of H-9 to C-3 (δ 69.8) and H-3 to C-4a (δ 111.2) (Figure 2 and Figure S16). In order to confirm the relative configuration, we calculated the coupling constant of H_2_-H_3_ and H_3_-H_4_ for the four possible relative configurations **3A**–**3D** (Figure 5). The computational ^3^*J* value of **3A** was most near to the measured result (Table 3). The absolute configuration was established by calculation of the specific rotation. The measured [α]_D_ value of **3** (−53.6) is consistent with the calculated one for (2*S*,3*S*,4*R*)-**3** (−100) and opposite to the calculated one for (2*R*,3*R*,4*S*)-**3** (+102). Thus, the structure of **3** was identified as (2*S*,3*S*,4*R*)-2-methylchroman-3,4,5-triol.

**Figure 6:**
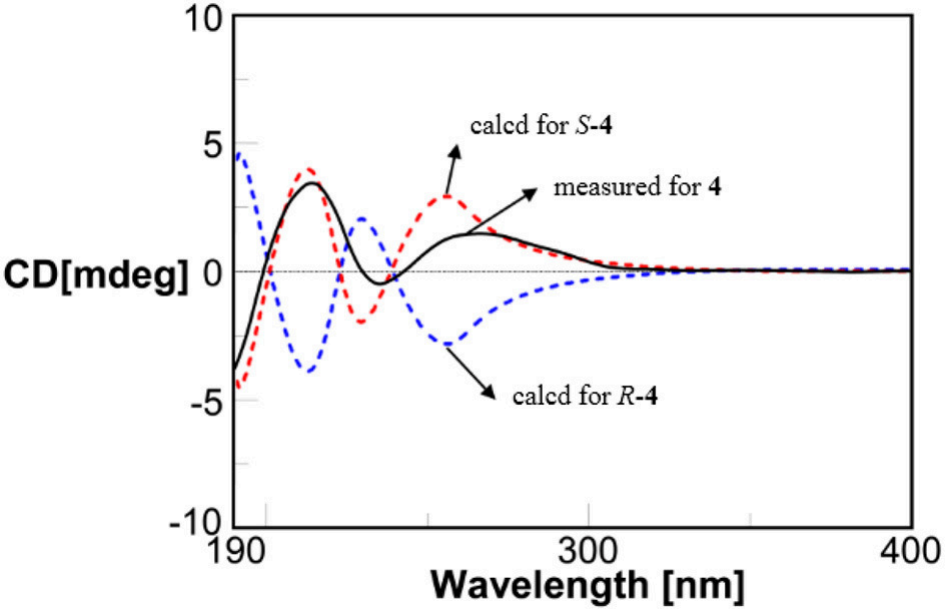
Measured and calculated ECD spectra for compound **4**.

Compound **4** showed the molecular formulae of C_11_H_14_O_5_ based on HRESIMS peaks at *m*/*z* 225.0767 [M–H]^−^ (calcd 225.0763), indicating five degrees of unsaturation. The 1D (Figures S17–S19) and HMQC (Figure S20) NMR spectra of **4** displayed three sp^2^ methines and four sp^2^ quaternary carbon signals, one sp^3^ oxygenated methine signals, one sp^3^ methylene signals and two methyl group (including one methoxy). The 1D NMR data (Table 1) of **4** were almost identical to those of **8** [Figure S1; (Dai et al., 2009)] except for an additional methoxy (δ_H/C_ 3.78/52.5) and the upfield shift for carbonyl carbon (−3.5 ppm), indicating that **4** is the methyl ester of **8**. This was confirmed by analysis of ^1^H-^1^H COSY correlation (Figure S21) and the key HMBC between the methoxy protons at δ_H_ 3.78 and the carbonyl carbon at δ_C−1_ 174.0 (Figure 2 and Figure S22). The specific rotations of both **4** ([α]_D_ +14.4) and **8** ([α]_D_ +8.2) were opposite to the synthetic analog, *R*-3-(3-methoxyphenyloxy)butanoic acid ([α]_D_ −31.2) (Kawasaki et al., 2008), indicating both **4** and **8** as *S*-configuration. The *S*-configuration of **4** was also backed by the coincidence of experimental and calculated ECD curves (Figure 6). Thus, the structure of compounds **4** and **8** were established as methyl (3*S*)-3-(2,3-dihydroxyphenyloxy) butanoate and (3*S*)-3-(2,3-dihydroxyphenyloxy)butanoic acid, respectively.

The molecular formula of compound **5** was determined as C_7_H_12_O_2_ based on the HREIMS peak at *m*/*z* 128.0845 [M]^+^ (calcd 128.0837), corresponding to two degrees of unsaturation. The IR spectrum showed hydroxy groups at 3442 cm^−1^ and double bonds at 3080 and 1646 cm^−1^. The 1D NMR spectra (Table 2, Figures S23–S25) of **5** showed two double bonds one of which is terminal, two oxygenated methines and one methyl group (Table 1). These groups were connected to the full structure of CH_2_ = CH–CH = CH–CH (OH)–CH (OH)–CH_3_ on the basis of ^1^H-^1^H COSY correlations from the methyl (δ_H_ 1.01) to the methylene (δ_H_ 5.03/5.17) through the two oxygenated methines (Figure 2 and Figure S26). The large value of ^3^*J*_H−4, H−5_ (15.4 Hz) corresponded to *E*-Δ^4^ double bond. The large ^3^*J*_H−2, H−3_ value (6.0 Hz) and the downfield methyl carbon signal (δ_C−1_ 19.1) indicated an *anti-*conformation (Jarvis et al., 1996; Zhang and O'doherty, 2005; Nilewski et al., 2009), corresponding to *threo*-configuration of 2,3-diol (Zheng et al., 2010). In order to confirm the relative configuration of compound **5**, ^3^*J*_H−2, H−3_ of *threo*-**5** and *erythro*-**5** were computed. The results showed that the predicted ^3^*J*_H−2, H−3_ values of *threo*-**5** (5.5 Hz) matched with the measured one (6.0 Hz) while the calculated one of *erythro*-**5** (3.8 Hz) was inconsistent, indicating *threo*- configuration. The direction of the specific rotation of **5** ([α]_D_ −6.8) were similar to the structurally related t-butyl (6*S*,7*S*)-6,7-dihydroxyocta-2,4-dienoate ([α]_D_ −23) (Zhang and O'doherty, 2005), and opposite to t-butyl (6*R*,7*R*)-6,7-dihydroxyocta-2,4-dienoate ([α]_D_ +22.9) (Zhang and O'doherty, 2005). The structure of **5** was thus deduced as (2*S*,3*S*,4*E*)-hepta-4,6-diene-2,3-diol.

**Table 2:** ^1^H (600 MHz) and ^13^C (150 MHz) NMR Data of Compounds **5** and **6** in DMSO-*d*_6_.

**Position**	**5**		**6^a^**	
	**δ_C_**	**δ_H_(*J* in Hz)**	**δ_C_**	**δ_H_(*J* in Hz)**
1	19.1, CH_3_	1.01 (d, 6.1)	61.9, CH_2_	3.65 (dt, 6.0, 2.2)
2	69.7, CH	3.46 (m)	36.0, CH_2_	2.31 (m)
3	75.1, CH	3.77 (dd, 6.0, 6.0)	129.4, CH	5.52 (dt, 15.4, 6.6)
4	130.2, CH	5.81 (dd, 15.4, 6.0)	130.2, CH	5.56 (dt, 15.4, 6.6)
5	136.1, CH	6.18 (dd, 15.4, 9.9)	40.1, CH_2_	2.15 (ddd, 14.3, 7.7, 6.6); 2.27 (m)
6	137.0, CH	6.34(ddd,17.0, 10.4, 9.9)	70.4, CH	3.69 (m)
7	116.2, CH_2_	5.03 (dd, 10.4, 1.7) 5.17 (dd, 17.0, 1.7)	40.2, CH_2_	2.24 (ddd,14.3, 7.7, 7.1) 2.31 (m)
8			130.5, CH	5.70 (dt, 14.8, 7.6)
9			134.2, CH	6.14 (dd, 14.8, 10.4)
10			136.9, CH	6.33 (ddd,17.0,10.4,10.4)
11			116.1, CH_2_	4.14 (d,17.0) 5.02 (d,10.4)

a *Measured in CDCl_3_*.

**Table 3:** The calculated ^3^*J*_H−2,H−3_ and ^3^*J*_H−3,H−4_ values of compound **3** for the four possible relative configurations.

	**H-2, H-3**		**H-3, H-4**	
	**Dihedral angle (°)**	**^3^*J* value (Hz)**	**Dihedral angle (°)**	**^3^*J* value (Hz)**
**3**		0.8		2.2
**3A**	66.2	3.1	83.8	1.4
**3B**	173.0	8.0	166.8	7.4
**3C**	59.2	3.9	42.6	5.8
**3D**	37.4	6.2	9.0	7.1

**Figure 7:**
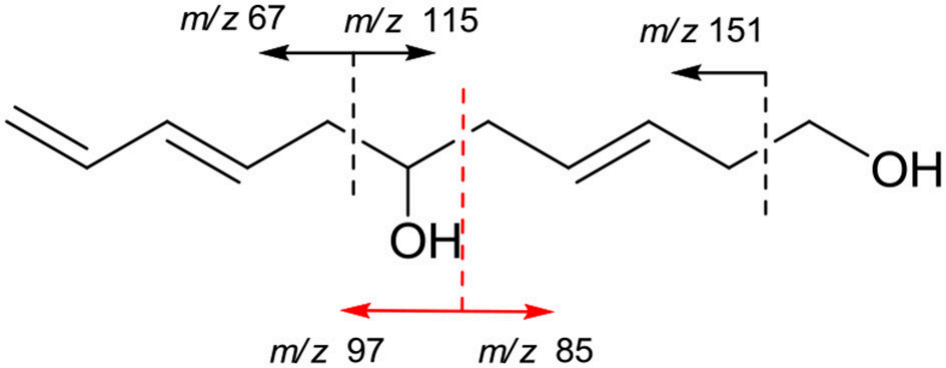
EI fragments of **6**.

The molecular formula of compound **6** was determined to be C_11_H_18_O_2_ based on the HREIMS peak at *m*/*z* 182.1305 [M]^+^ (calcd. 182.1307), indicating three degrees of unsaturation. The EIMS of **6** illustrated in Figure 7 indicates the existence of -CH_2_OH, -C_6_H_9_O, and -C_5_H_9_O moieties. The ^1^H (Figure S27) and ^13^C (Figure S28) NMR spectra and DEPT (Figure S29) and HMQC (Figure S30) experiments of **6** revealed 11 signals including three double bonds one of which is terminal, one oxygenated methine, four methylenes one of which is oxygenated. The ^1^H-^1^H COSY (Figure S31) correlations from H-1 (δ 3.65) to H-11 (δ 4.14/5.02) in sequence established the structure, CH_2_ = CH–CH = CH–CH_2_-CH(OH)–CH_2_-CH = CH–CH_2_-CH_2_OH, which was supported by HMBC correlations (Figure S32). The large values of ^3^*J*_H−3, H−4_ (15.4 Hz) and ^3^*J*_H−8, H−9_ (14.8 Hz) suggested that both Δ^3^ and Δ^8^ double bonds were *E-*configurations. The direction of specific rotation of **6** ([α]_D_ +2.0) is similar to that of (*S*)-dodeca-3,5-diene-1,7-diol ([α]_D_ +56) (Zhang and Kyler, 1989), suggesting *S*-configuration at C-6. Thus, the structure of **6** was deduced as (3*E*,8*E*,6*S*)-undeca-3,8,10-triene-1,6-diol.

**Figure 8:**
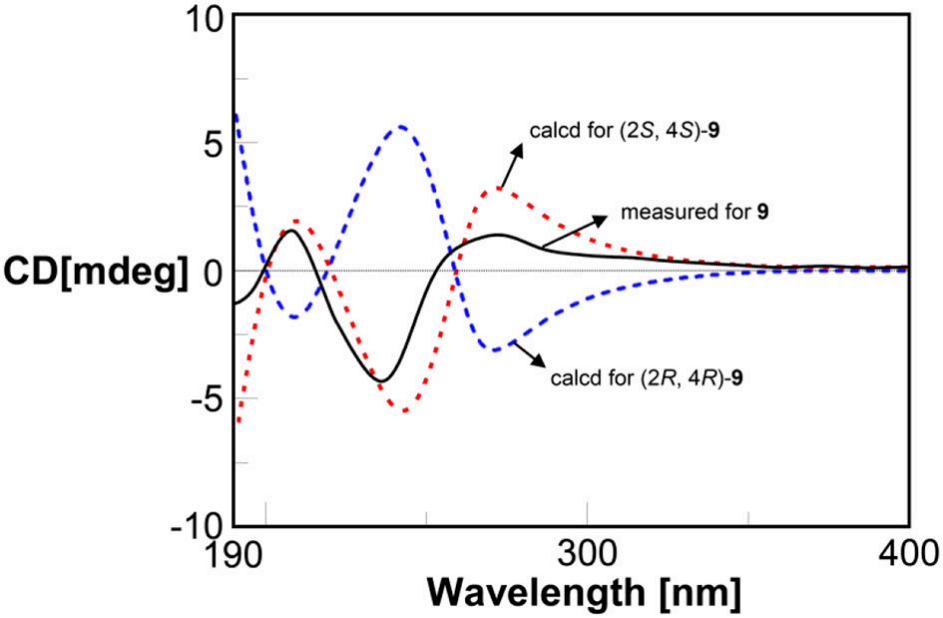
Measured and calculated ECD spectra for compound **9**.

The relative configurations of compounds **9** and **10** were determined as (–)-*trans*-4-methoxy-2-methylchroman-5-ol (Wu et al., 2010) and (–)-*trans*-2-methyl chroman-4,5 -diol (Teles et al., 2005), respectively. The absolute configuration of compound **9** was determined by quantum chemical ECD calculation. The measured ECD of **9** was coincident with the calculated ECD of (2*S*,4*S*)-**9** and opposite to ECD of (2*R*,4*R*)-**9** (Figure 8). Thus, compound **9** was established to be (2*S*,4*S*)-4-methoxy-2-methyl chroman-5-ol. The similar sign of the specific rotations of **9** and **10** (${\left[\alpha \right]}_{\text{D}}^{22}$ −2.0 vs. ${\left[\alpha \right]}_{\text{D}}^{22}$ −6.0, MeOH) suggests the same absolute configuration. Therefore, compound **10** was determined to be (2*S*,4*S*)-2-methylchroman-4,5-diol. The absolute configurations of compounds **9** and **10** were determined for the first time in this study.

(2*R*)-7-*O*-α-D-Ribofuranosyl-5-hydroxy-2-methylchroman-4-one (**1**): White amorphous powder; ${\left[\alpha \right]}_{\text{D}}^{23}$ +198.9 (*c* 0.1, MeOH); UV (MeOH) λ_max_ (log ε) 204 (3.62), 278 (3.55), 320 (2.77) nm; ECD (MeOH) λ_max_ (Δε) 211 (+14.21), 284 (−3.51), 327 (+3.42); IR (KBr) ν_max_ 3,416, 1,646, 1,573, 1,354, 1,295, 1,195, 1,155, 1,076, 1,029 cm^−1^; ^1^H and ^13^C NMR (Table 1); HRESIMS *m*/*z* 327.1068 [M+H]^+^ (calcd for C_15_H_19_O_8_ 327.1080).

(2*S*)-7-*O*-α-D-Ribofuranosyl-5-hydroxy-2-methylchroman-4-one (**2**): White amorphous powder; ${\left[\alpha \right]}_{\text{D}}^{23}$ +118.6 (*c* 0.1, MeOH); UV (MeOH) λ_max_ (log ε) 204 (3.62), 278 (3.55), 320 (2.77) nm; ECD (MeOH) λ_max_ (Δε) 211 (−10.04), 284 (+9.40), 330 (−2.13); IR (KBr) ν_max_ 3,416, 1,646, 1,573, 1,354, 1,295, 1,195, 1,155, 1,076, 1,029 cm^−1^; ^1^H and ^13^C NMR (Table 1); HRESIMS *m*/*z* 327.1068 [M+H]^+^ (calcd for C_15_H_19_O_8_ 327.1080).

(2*S*,3*S*,4*R*)-2-Methylchroman-3,4,5-triol (**3**): Colorless oil; ${\left[\alpha \right]}_{\text{D}}^{23}$ −53.6 (*c* 0.1, MeOH); UV (MeOH) λ_max_ (log ε) 200 (3.25), 270 (2.26) nm; IR (KBr) ν_max_ 3,429, 2,356, 1,627, 1,4,01, 1,090 cm^−1^; ^1^H and ^13^C NMR (Table 1); HRESIMS *m*/*z* 195.0659 [M–H] ^−^ (calcd. for C_10_H_11_O_4_: 195.0657).

Methyl (3*S*)-3-(2,3-dihydroxyphenyloxy)butanoate (**4**): Colorless oil; ${\left[\alpha \right]}_{\text{D}}^{23}$ +14.4 (*c* 0.1, MeOH); UV (MeOH) λ_max_ (log ε) 200 (3.32), 270 (2.19) nm; IR (KBr) ν_max_ 3,409, 2,356, 1,706, 1,606, 1,481, 1,202, 1,063, 1,010 cm^−1^; ^1^H and ^13^C NMR (Table 1); HRESIMS *m*/*z* 225.0767 [M–H]^−^(calcd. for C_11_H_13_O_5_ 225.0763)

(2*S*,3*S*,4*E*)-Hepta-4,6-diene-2,3-diol (**5**): Colorless oil; ${\left[\alpha \right]}_{\text{D}}^{23}$ −6.8 (*c* 0.1, MeOH); UV (MeOH) λ_max_ (log ε) 200 (3.09), 217 (3.38) nm; IR (KBr) ν_max_ 3442, 3080, 1646, 1540, 1023, 446 cm^−1^; ^1^H and ^13^C NMR (Table 2); EIMS *m/z* (%): 129 (45), 256 (8), 111 (26), 97 (51), 83 (69), 82 (38); HREIMS *m*/*z* 128.0845 [M]^+^ (calcd. for C_7_H_12_O_2_ 128.0837).

(3*E*,8*E*,6*S*)-Undeca-3,8,10-trien-1,6-diol (**6**): Colorless oil; ${\left[\alpha \right]}_{\text{D}}^{23}$ +2.0 (*c* 0.1, MeOH); UV (MeOH) λ_max_ (log ε) 200 (2.86), 218 (3.16) nm; IR (KBr) ν_max_ 3,390, 2,927, 1,715, 1,421, 1,047, 973 cm^−1^; ^1^H and ^13^C NMR (Table 2); EIMS *m/z* (%): 181 (8), 164 (9), 151 (14), 129 (17), 115 (16), 97 (31), 85 (28), 71 (53), 67(88), 53(10); HREIMS *m*/*z* 182.1305 [M]^+^ (calcd. for C_11_H_18_O_2_ 182.1307).

### X-ray crystallographic data of 1

Compound **1** was obtained as a colorless monoclinic crystal with molecular formula of C_15_H_18_O_8_ from MeOH and H_2_O. Space group *P*2_1_, *a* = 7.0121(7) Å, *b* = 10.6659(11) Å, *c* = 9.8560(8) Å, α = 90.00°, β = 95.3230(10)°, γ = 90.00°, *V* = 733.95(12) Å^3^, *Z* = 2, *D*_calcd_ = 1.476 mg/m^3^, μ = 0.121 mm^−1^, *F*(000) = 344, crystal size 0.42 ×0.30 ×0.21 mm. A total of 3413 unique reflections (2θ<50°= were collected on a CCD area detector diffractometer with graphite monochromated Mo-Ka radiation (λ = 0.71073 Å). The structure was solved by direct methods (SHELXS-97) and expanded using Fourier techniques (SHELXL-97). The final cycle of full-matrix least squares refinement was based on 2053 unique reflections (2θ <50°) and 210 variable parameters and converged with unweighted and weighted agreement factors of *R*_1_ = 0.0421, *R*_w_ = 0.0981 and *R* = 0.0374 for *I*>*2sigma(I)* data. Crystallographic data (excluding structure factors) for structure **1** in this paper have been deposited in the Cambridge Crystallographic Data Centre as supplementary publication number CCDC 883328 [fax: +44 (0)-1223-336033 or e-mail: deposit@ccdc.cam.ac.uk].

### Biogenetic origin

**Figure 9:**
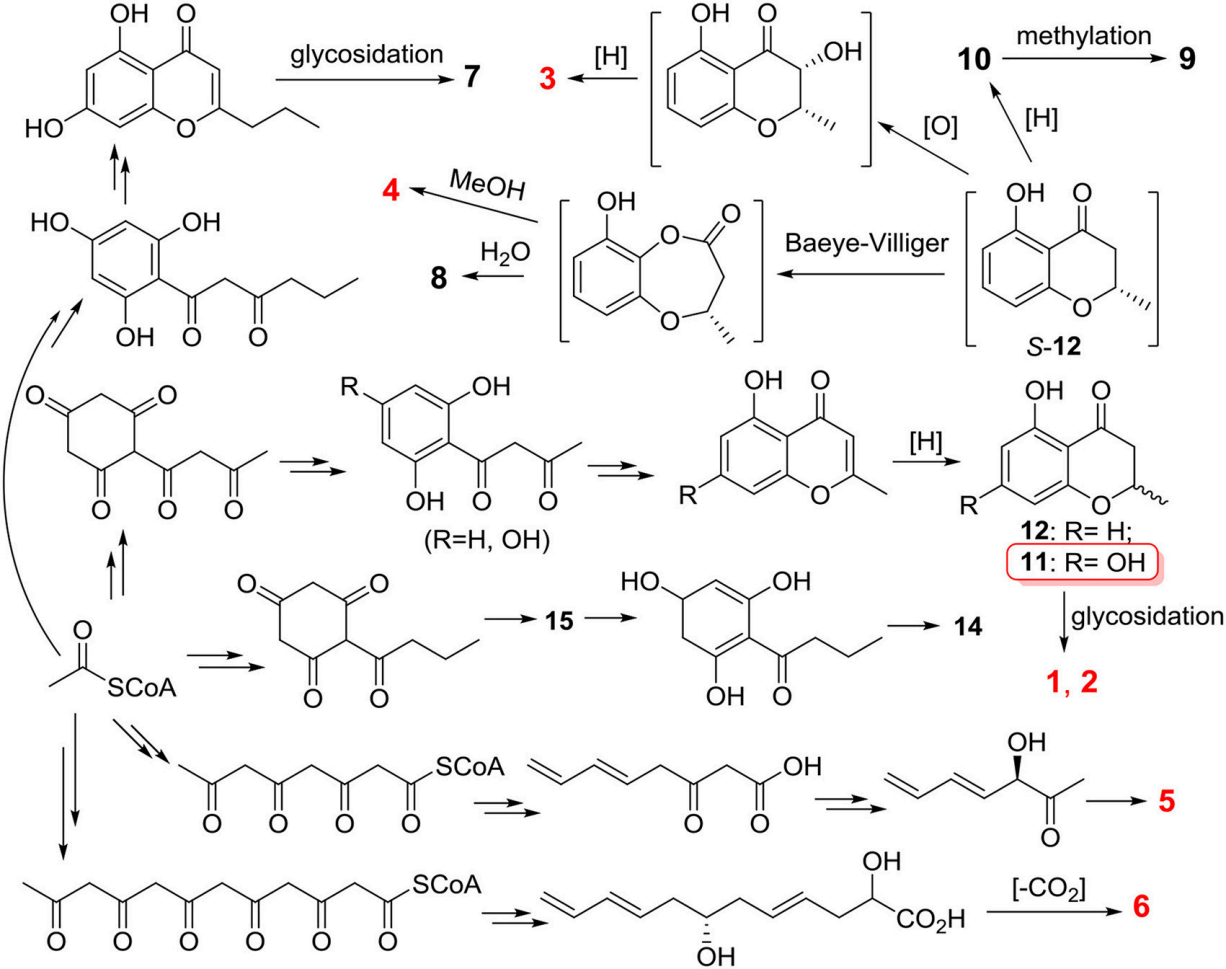
Possible biosynthetic pathway of the compounds **1**–**15**.

These compounds were postulated to be biosynthesized by the polyketide pathway from acetyl coenzyme A (Figure 9). The acetyl-CoA units underwent condensation, cyclization, dehydration and hydrogenation to produce compounds **11** and **12**. Compound **11** formed compounds **1** and **2** by glycosidation. (*S*)-**12** underwent oxidation and reduction to yield compound **3**. The reduction of (*S*)-**12** produced compound **10** that was transformed to compound **9** followed by methylation. (*S*)-**12** was subjected to Baeyer-Villiger oxidation followed by methanolysis and hydrolysis to yield compounds **4** and **8**, respectively. Compounds **5** and **6** were formed from different lengths of acetyl-CoA units by condensation, reduction, dehydration, and decarboxylation. The condensation of acetyl-CoA units followed by cyclization and reduction formed compound **15** that was transformed to compound **14** after enolization and dehydration.

### Biological activity

Compounds **1**–**14** were tested for cytotoxic effects on the HL-60, BEL-7402, K562, A549, HeLa, and H1975 cell lines, DPPH scavenging activity, and antimicrobial activities against *E. coli, E. aerogenes, P. aeruginosa, B. subtilis*, and *C. albicans*. As the results, compound **6** was cytotoxic to H1975 cell line with an IC_50_ values of 10.0 μM, while compounds **4** and **8**–**10** showed DPPH radical scavenging activity with the IC_50_ values of 2.65, 0.24, 5.66, and 6.67 μM, respectively. None of the compounds exhibit antimicrobial activities.

## Conclusions

Five new polyketides were isolated and identified from the fermentation of the mangrove fungus *Cladosporium* sp. OUCMDZ-302 with *Excoecaria agallocha*. The new compound **4** showed DPPH radical scavenging activity with an IC_50_ value of 2.65 μM.

## Author contributions

All authors listed have made a substantial, direct and intellectual contribution to the work, and approved it for publication.

### Conflict of interest statement

The authors declare that the research was conducted in the absence of any commercial or financial relationships that could be construed as a potential conflict of interest.
