# Supplementary material for: Polyketides From the Endophytic Fungus Cladosporium sp. Isolated From the Mangrove Plant Excoecaria agallocha
Source: Front Chem. 2018 Aug 14;6:344. doi: 10.3389/fchem.2018.00344 (PMC6102696; doi:10.3389/fchem.2018.00344)
Supplement: Supplementary file 1 [file Data_Sheet_1.docx]

Supplementary Material

Polyketides from the Endophytic Fungus *Cladosporium* sp. isolated from the Mangrove Plant *Excoecaria agallocha*

Liping Wang^1,2†^, Xiuli Han^2,3†^, Guoliang Zhu^2^, Yi Wang^2^, Arthit Chairoungdua^5^, Pawinee Piyachaturawat^5^ and Weiming Zhu^1,2,4*^

^1^ State Key Laboratory of Functions and Applications of Medicinal Plants, Guizhou Medical University, Guiyang 550014, China

^2^ Key Laboratory of Marine Drugs, Ministry of Education of China, School of Medicine and Pharmacy, Ocean University of China, Qingdao 266003, China

^3^ Colleage of Life Science, Shandong University of Technology, Zibo 255049, Shandong, China

^4^ Laboratory for Marine Drugs and Bioproducts of Qingdao National Laboratory for Marine Science and Technology; Qingdao 266003, China

^5^ Department of Physiology, Faculty of Science, Mahidol University, Bangkok 10400, Thailand

^†^ These authors contributed equally to this work

***Correspondence:**Weiming Zhu
weimingzhu@ouc.edu.cn

**List of Supporting Information**

**Figure S1**. The ^1^H-NMR spectrum of mixture **1** and **2** in DMSO-*d*_6_………………………………S3

**Figure S2**. The ^13^C-NMR spectrum of mixture **1** and **2** in DMSO-*d*_6_…………………………………S4

**Figure S3**. The DEPT spectrum of mixture **1** and **2** in DMSO-*d*_6_…………………………………….S5

**Figure S4**. The HMQC spectrum of mixture **1** and **2** in DMSO-*d*_6_…………………………………...S6

**Figure S5**. The ^1^H-^1^H COSY spectrum of mixture **1** and **2** in DMSO-*d*_6_……………………………..S7

**Figure S6**. The HMBC spectrum of mixture **1** and **2** in DMSO-*d*_6_……………………………………S8

**Figure S7**. The ^1^H-NMR spectrum of compound **1** in DMSO-*d*_6_………………………………….S9

**Figure S8**. The ^13^C-NMR spectrum of compound **1** in DMSO-*d*_6_…………………………………S10

**Figure S9**. The ^1^H-NMR spectrum of compound **2** in DMSO-*d*_6_……………………………………S11

**Figure S10**. The ^13^C-NMR spectrum of compound **2** in DMSO-*d*_6_…………………………………S12

**Figure S11**. The ^1^H-NMR spectrum of compound **3** in DMSO-*d*_6_….........................……….....…..S13

**Figure S12**. The ^13^C-NMR spectrum of compound **3** in DMSO-*d*_6_…....……………......…………..S14

**Figure S13**. The DEPT spectrum of compound **3** in DMSO-*d*_6_…. .……........................................…S15

**Figure S14**. The HMQC spectrum of compound **3** in DMSO-*d*_6_……..………………………...…..S16

**Figure S15**. The ^1^H-^1^H COSY spectrum of compound **3** in DMSO-*d*_6_…………………………….. .S17

**Figure S16**. The HMBC spectrum of compound **3** in DMSO-*d*_6_........….…………………...……..S18

**Figure S17**. The ^1^H-NMR spectrum of methyl compound **4** in CDCl_3_………………………………S19

**Figure S18**. The ^13^C-NMR spectrum of methyl compound **4** in CDCl_3_…………………………...S20

**Figure S19**. The DEPT spectrum of compound **4** in CDCl_3_………………………………....………S21

**Figure S20**. The HMQC spectrum of compound **4** in CDCl_3_…………………………………..……S22

**Figure S21**. The ^1^H-^1^H COSY spectrum of compound **4** in CDCl_3_………………………………….S23

**Figure S22**. The HMBC spectrum of compound **4** in CDCl_3_…………………………………..……S24

**Figure S23**. The ^1^H-NMR spectrum of compound **5** in DMSO-*d*_6_…….……………………….……S25

**Figure S24**. The ^13^C-NMR spectrum of compound **5** in DMSO-*d*_6_…….………………….…….. S26

**Figure S25**. The DEPT spectrum of compound **5** in DMSO-*d*_6_……………………………….….. S27

**Figure S26**. The ^1^H-^1^H COSY spectrum of compound **5** in DMSO-*d*_6_………………………….. S28

**Figure S27**. The ^1^H-NMR spectrum of compound **6** in CDCl_3_…………………………………….S29

**Figure S28**. The ^13^C-NMR spectrum of compound **6** in CDCl_3_…………………………………….S30

**Figure S29**. The DEPT spectrum of compound **6** in CDCl_3_……………………………………….S31

**Figure S30**. The HMQC spectrum of compound **6** in CDCl_3_…………………….……………….S32

**Figure S31**. The ^1^H-^1^H COSY spectrum of compound **6** in CDCl_3_……………………………….S33

**Figure S32**. The HMBC spectrum of compound **6** in CDCl_3_……………………………………….S34

**Figure S33**. GC-MS of D- and L-ribose derivative…………………………...…...………..……….S35

**Figure S34**. GC-MS of the derivative of sugar from mixture of **1** and **2** …………………………….S36

**Figure S35**. The sporogenous structure of *Cladosporium* sp. OUCMDZ-302………………………S37

**Figure S36**. The structures and physical properties of compounds **7**–**15**.…………..………………S37

**Table S1** NMR data of compounds **7**–**11**……………………………...…………………………….S39

**Figure S1**. The ^1^H-NMR spectrum of the mixture of **1** and **2** in DMSO-*d*_6_

**Figure S2**. The ^13^C-NMR spectrum of the mixture of **1** and **2** in DMSO-*d*_6_

**Figure S3**. The DEPT spectrum of the mixture of **1** and **2** in DMSO-*d*_6_

**Figure S4**. The HMQC spectrum of the mixture of **1** and **2** in DMSO-*d*_6_

**Figure S5**. The ^1^H-^1^H COSY spectrum of the mixture of **1** and **2** in DMSO-*d*_6_

**Figure S6**. The HMBC spectrum of the mixture of **1** and **2** in DMSO-*d*_6_

**Figure S7**. The ^1^H-NMR spectrum of compound **1** in DMSO-*d*_6_

**Figure S8**. The ^13^C-NMR spectrum of compound **1** in DMSO-*d*_6_

**Figure S9**. The ^1^H-NMR spectrum of compound **2** in DMSO-*d*_6_

**Figure S10**. The ^13^C-NMR spectrum of compound **2** in DMSO-*d*_6_

**Figure S11**. The ^1^H-NMR spectrum of compound **3** in DMSO-*d*_6_

**Figure S12**. The ^13^C-NMR spectrum of compound **3** in DMSO-*d*_6_

**Figure S13**. The DEPT spectrum of compound **3** in DMSO-*d*_6_

**Figure S14**. The HMQC spectrum of compound **3** in DMSO-*d*_6_

**Figure S15**. The ^1^H-^1^H COSY spectrum of compound **3** in DMSO-*d*_6_

**Figure S16**. The HMBC spectrum of compound **3** in DMSO-*d*_6_

**Figure S17**. The ^1^H-NMR spectrum of compound **4** in CDCl_3_

**Figure S18**. The ^13^C-NMR spectrum of compound **4** in CDCl_3_

**Figure S19**. The DEPT spectrum of compound **4** in CDCl_3_

**Figure S20**. The HMQC spectrum of compound **4** in CDCl_3_

**Figure S21**. The ^1^H-^1^H COSY spectrum of compound **4** in CDCl_3_

**Figure S22**. The HMBC spectrum of methyl compound **4** in CDCl_3_

**Figure S23**. The ^1^H-NMR spectrum of compound **5** in DMSO-*d*_6_

**Figure S24**. The ^13^C-NMR spectrum of compound **5** in DMSO-*d*_6_

**Figure S25**. The DEPT spectrum of compound **5** in DMSO-*d*_6_

**Figure S26**. The^1^H-^1^H COSY spectrum of compound **5** in DMSO-*d*_6_

**Figure S27**. The ^1^H-NMRspectrum of compound **6** in CDCl_3_

**Figure S28**. The ^13^C-NMR spectrum of compound **6** in CDCl_3_

**Figure S29**. The DEPT spectrum of compound **6** in CDCl_3_

**Figure S30**. The HMQC spectrum of compound **6** in CDCl_3_

**Figure S31**. The ^1^H-^1^H COSY spectrum of compound **6** in CDCl_3_

**Figure S32**. The HMBC spectrum of compound **6** in CDCl_3_

**Figure S33**. GC-MS diagrams of D- and L-ribose derivatives

D-ribose derivative

L-ribose derivative

Mixture of D- and L-ribose derivative

**Figure S34**. GC-MS diagrams of the derivatives of sugar from the mixture of **1** and **2**

Derivatives of sugar from the mixture of **1** and **2**

Mixture of derivatives of sugar from **1** and **2** with D- and L-ribose derivatives

**Figure S35**. The sporogenous structure of *Cladosporium* sp. OUCMDZ-302


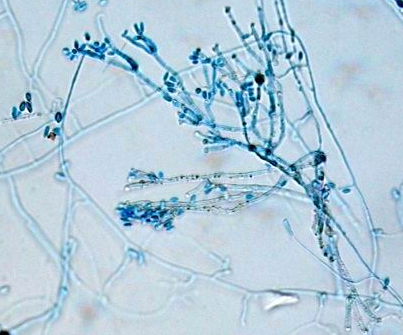

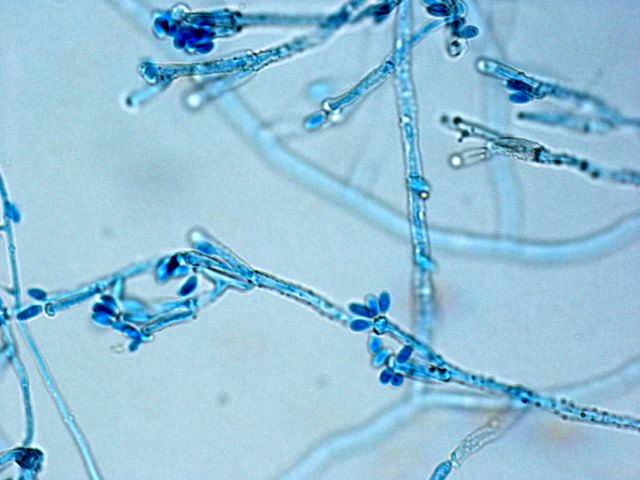


(40×10) (100×10)

**Figure S36**. The structures and physical properties of compounds **7**–**15**

**7-*O*-α-D-Ribofuranosyl-5-hydroxy-2-propylchromen-4-one (7)**: colorless oil; [α]^23^_D_ +90.4 (*c* 0.1, MeOH); UV (MeOH) *λ*_max_ (log *є*) 200 (3.53), 220 (3.51), 240 (3.57), 247 (3.56), 282 (3.15), 311 (2.95) nm; IR (KBr) ν_max_ 3337, 2965, 2925, 1660, 1613, 1573, 1487, 1414, 1321, 1275, 1164, 1040, 950, 844, 751 cm^−1^; ^1^H and ^13^C NMR data (see Table S1); HRESIMS *m*/*z* 353.1224 [M +H]^+^ (calcd for C_17_H_21_O_8_ 353.1236).

**(3*S*)-3-(2,3-Dihydroxyphenyloxy)butanoic acid (8)**: colorless oil; [α]^23^_D_ +8.2 (*c* 0.1, MeOH); UV (MeOH) *λ*_max_ (log *є*) 200 (3.32), 270 (2.19) nm; IR (KBr) *ν*_max_ 3317, 1706, 1606, 1474, 1288, 1202, 1069, 1010, 765, 725 cm^-1^; ^1^H and ^13^C NMR data (see Table S1); HRESIMS *m/z* 211.0610 [M–H]^–^  (calcd. for C_10_H_11_O_5_ 211.0606).

**(2*S*,4*S*)-4-Methoxy-2-methylchroman-5-ol (9)**: colorless oil; [α]^22^_D_ –16.1 (*c* 0.06, CHCl_3_), –2.0 (*c* 0.05, MeOH); UV (MeOH) *λ*_max_ (log *є*) 200 (3.21), 270 (2.28) nm; IR (KBr) *ν*_max_ 3317, 2965, 2925, 1613, 1586, 1467, 1348, 1288, 1222, 1085, 1010, 917, 778, 731cm^-1^; EIMS *m/z* (%): 194 (M^+^, 5), 193 (23), 163 (7), 166 (90), 147 (33), 123 (100), 95 (13); ^1^H and ^13^C NMR data (see Table S1); ESIMS *m*/*z* 192.9 [M–H]^–^.

**(2*S*,4*S*)-2-methylchroman-4,5-diol (10)**: colorless oil; [α]^22^_D_ –6.0 (*c* 0.05, MeOH); ^1^H and ^13^C NMR data (see Table S1); ESIMS *m*/*z* 178.9 [M–H]^–^.

**(±)-5,7-dihydroxy-****2-methylchroman-4-one (11)**: yellowish oil; [α]^22^_D_ 0 (*c* 0.1, MeOH); ^1^H and ^13^C NMR data (see Table S1); ESIMS *m*/*z* 193.0 [M–H]^–^.

**(±)-5-hydroxy-2-methylchroman-4-one (12)**: yellowish amorphous powder; [α]^22^_D_ 0 (*c* 0.1, MeOH); ^1^H (600 MHz, CDCl_3_) *δ*_H_ 4.54 (m, 1H, H-2), 2.66 (dd, 1H, *J*= 3.6, 17.4 Hz, H-3a), 2.71 (dd, 1H, *J*= 11.8, 17.4 Hz, H-3b); 6.46 (dd, 1H, *J*= 8.2, 1.1 Hz, H-6), 7.33 (dd, 1H, *J*= 8.2, 8.2, H-7), 6.40 (dd, 1H, *J*= 8.2, 1.1 Hz, H-8), 1.50 (d, 3H, *J*= 6.3 Hz, H-9); ^13^C-NMR (150MHz, CDCl_3_) *δ*_C_ 73.9 (CH, C-2), 43.8 (CH_2_, C-3), 198.6 (C, C-4), 108.1 (C, C-4a), 162.1 (C, C-5); 109.2 (CH, C-6), 138.2 (CH, C-7), 107.3 (CH, C-8), 161.7 (C, C-8a), 20.9 (CH_3_, C-9); ESIMS *m*/*z* 177.1 [M–H]^–^.

**1-(2,6-dihydroxyphenyl)ethanone (13)**: yellowish amorphous powder; ^1^H (600 MHz, CDCl_3_) *δ*_H_ 2.64 (s, 3H, H-2), 6.39 (d, 2H, *J*= 8.2 Hz, H-3'/5'), 7.26 (dd, 1H, *J*= 8.2, 8.2 Hz, H-4'); ESIMS *m*/*z* 151.1 [M–H]^–^.

**1-(2,6-dihydroxyphenyl)-1-butanone (14)**: yellowish amorphous powder; ^1^H (600 MHz, CDCl_3_) *δ*_H_ 3.12 (t, 2H, *J*= 7.7 Hz, H-2), 1.74 (m, 2H, H-3), 1.00 (t, 3H, *J*= 7.7 Hz, H-4), 6.39 (d, 2H, *J*= 7.7 Hz, H-3'/5'), 7.23 (dd, 1H, *J*= 7.7, 7.7 Hz, H-4'); ESIMS *m*/*z* 179.1 [M–H]^–^.

**(±)-2-Butyryl-3,5-dihydroxycyclohex-2-enone (15)**: colorless oil; [α]^22^_D_ 0 (*c* 0.5, CHCl_3_); ^1^H-NMR (600 MHz, CDCl_3_) *δ*_H_ 2.74 (dd, 1H, *J*=3.2, 16.7 Hz, H-4a), 2.63 (dd, 1H, *J*= 5.8, 16.7 Hz, H-4b), 4.40 (m, 1H, H-5), 2.94 (dd, 1H, *J*= 3.8, 17.9 Hz, H-6a), 2.80 (dd, 1H, *J*= 5.8, 17.9 Hz, H-6b), 2.99 (t, 2H, *J*= 7.7, 7.0 Hz, H-8), 1.65 (m, 2H, H-9), 0.98 (t, 3H, t, *J*= 7.1, 7.7 Hz, H-10), 18.36 (s, 1H, HO-3); ^13^C NMR (150 MHz, CDCl_3_) *δ*_C_ 196.5 (C, C-1), 113.0 (C, C-2), 193.7 (C, C-3), 47.2 (CH_2_, C-4), 63.3 (CH, C-5), 41.5 (CH_2_, C-6), 205.5 (C, C-7), 42.3 (CH_2_, C-8), 18.0 (CH_2_, C-9), 14.0 (CH_3_, C-10); ESIMS *m*/*z* 197.1 [M–H]^–^.

**Table S1.** NMR data of compounds **7**–**11**

| Position | **7** (DMSO-*d*_6_) | | | **8** (CDCl_3_) | | **9** (DMSO-*d*_6_) | | **10** (CDCl_3_) | | **11** (DMSO-*d*_6_) | |
| --- | --- | --- | --- | --- | --- | --- | --- | --- | --- | --- | --- |
|  | *δ*_C_ | *δ*_H_ (*J* in Hz) | | *δ*_C_ | *δ*_H_ (*J* in Hz) | *δ*_C_ | *δ*_H_ (*J* in Hz) | *δ*_H_ (*J* in Hz) | | *δ*_C_ | *δ*_H_ (*J* in Hz) |
| 1 |  | |  | 177.5, C |  |  |  |  | |  |  |
| 2 | 171.0, C | |  | 40.8, CH_2_ | 2.82 (dd, 9.2, 16.5);  2.66 (d, 16.5) | 66.8, CH | 4.15 (m) | 4.16 (m) | | 73.7, CH | 4.56 (m) |
| 3 | 107.8, CH | | 6.24 (s) | 73.1, CH | 4.5 (m) | 33.3, CH_2_ | 1.43 (ddd, 14.3, 12.1, 3.3); 2.10 (ddd, 14.3, 2.2, 1.8) | 1.85 (ddd, 14.2, 14.2, 3.3) 2.10 (ddd, 14.2, 3.3, 3.3) | | 42.9, CH_2_ | 2.76 (dd, 12.4, 17.4) 2.59 (dd, 3.2, 17.4) |
| 4 | 182.0, C | |  | 19.6, CH_3_ | 1.33 ( d, 6.0) | 66.8, CH | 4.38 (dd, 3.3, 2.2) | 5.00 (dd, 3.3, 3.3 ) | | 196.4, C |  |
| 4a | 104.9, C | |  |  |  | 109.8, C |  |  |  | 108.1, C |  |
| 5 | 157.4, C | |  |  |  | 156.9, C |  |  |  | 163.5, C |  |
| 6 | 94.7, CH | | 6.65 (d, 2.3) |  |  | 106.3, CH | 6.34 (dd, 8.0, 1.1) | 6.43 (dd, 7.7, 1.3) | | 95.6, CH | 5.84 (s) |
| 7 | 162.8, C | |  |  |  | 129.1, CH | 6.95 (dd, 8.5, 8.0) | 7.10 (dd, 8.8, 7.7) | | 166.6, C |  |
| 8 | 99.9, CH | | 6.41 (d, 2.3) |  |  | 106.8, CH | 6.21 (dd, 8.5, 1.1) | 6.48 (dd, 8.8, 1.3) | | 94.8, CH | 5.84 (s) |
| 8a | 161.0, C | |  |  |  | 155.7, C |  |  |  | 162.9, C |  |
| 9 | 34.5,CH_2_ | | 2.63 (t, 7.3) |  |  | 21.1, CH_3_ | 1.33 (d, 6.6) | 1.44 (d, 5.5) | | 20.3, CH_3_ | 1.38 (d, 6.3) |
| 10 | 19.6, CH_2_ | | 1.69 (tq, 7.3, 7.3) |  |  | 55.7, CH_3_ | 3.33 (s) |  |  |  |  |
| 11 | 13.2, CH_3_ | | 0.95 (t, 7.3) |  |  |  |  |  |  |  |  |
| 1’ | 100.2, CH | | 5.74 (d, 4.6) | 144.2, C |  |  |  |  |  |  |  |
| 2’ | 71.5, CH | | 4.10(m) | 136.0, C |  |  |  |  |  |  |  |
| 3’ | 69.2, CH | | 3.93 (m) | 145.1, C |  |  |  |  |  |  |  |
| 4’ | 86.7, CH | | 3.98 (m) | 110.9, CH | 6.70 (d, 7.8, ) |  |  |  |  |  |  |
| 5’ | 61.4, CH | | 3.47 (m) | 119.6, CH | 6.68 (dd, 7.8, 7.3) |  |  |  |  |  |  |
| 6’ |  | |  | 111.6, CH | 6.52 (d, 7.3) |  |  |  |  |  |  |
